# Population-Based Clinical Cancer Registration in Germany

**DOI:** 10.3390/cancers15153934

**Published:** 2023-08-02

**Authors:** Alexander Katalinic, Marco Halber, Martin Meyer, Maren Pflüger, Andrea Eberle, Alice Nennecke, Soo-Zin Kim-Wanner, Tobias Hartz, Kerstin Weitmann, Andreas Stang, Christina Justenhoven, Bernd Holleczek, Daniela Piontek, Ian Wittenberg, Annika Heßmer, Klaus Kraywinkel, Claudia Spix, Ron Pritzkuleit

**Affiliations:** 1 Cancer Registry Schleswig-Holstein, 23562 Lübeck, Germany; ron.pritzkuleit@uksh.de; 2 Institute for Social Medicine and Epidemiology, University of Lübeck, 23562 Lübeck, Germany; 3 Cancer Registry Baden-Wurttemberg, 70191 Stuttgart, Germany; halber@klr-krbw.de; 4 Bavarian Cancer Registry, 90441 Nurnberg, Germany; martin.meyer@lgl.bayern.de; 5 Cancer Registry Brandenburg-Berlin, 03048 Cottbus, Germany; maren.pflueger@kkrbb.de; 6 Cancer Registry Bremen, 28359 Bremen, Germany; eberle@leibniz-bips.de; 7 Hamburg Cancer Registry, 20097 Hamburg, Germany; alice.nennecke@bwfgb.hamburg.de; 8 Hessian Cancer Registry, 60439 Frankfurt, Germany; soo-zin.kim-wanner@hlpug.hessen.de; 9 Cancer Registry Lower Saxony, 30659 Hannover, Germany; t.hartz@kk-n.de; 10 Cancer Registry Mecklenburg-Western Pomerania, 17475 Greifswald, Germany; kerstin.weitmann@uni-greifswald.de; 11 Cancer Registry North Rhine-Westphalia, 44801 Bochum, Germany; andreas.stang@krebsregister.nrw.de; 12 Cancer Registry Rhineland-Palatinate, 55116 Mainz, Germany; justenhoven@krebsregister-rlp.de; 13 Saarland Cancer Registry, 66119 Saarbrucken, Germany; b.holleczek@krebsregister.saarland.de; 14 Joint Office of the Clinical Cancer Registries in Saxony, 01099 Dresden, Germany; d.piontek@slaek.de; 15 Cancer Registry Saxony-Anhalt, 06112 Halle (Saale), Germany; i.wittenberg@kkr-lsa.de; 16 Cancer Registry Thuringia, 07743 Jena, Germany; annika.hessmer@zkkr-thueringen.de; 17 Centre for Cancer Registry Data at the Robert Koch-Institute, 12101 Berlin, Germany; kraywinkelk@rki.de; 18 Division of Childhood Cancer Epidemiology, German Childhood Cancer Registry, 55101 Mainz, Germany; clauspix@uni-mainz.de

**Keywords:** cancer registration, cancer, Germany, incidence, quality assurance, benchmarking

## Abstract

**Simple Summary:**

Cancer registration has a long tradition in Germany. In 2013, new legislation obliged all German states to implement additional clinical cancer registration, including standardised documentation of all therapies, recurrences and further follow-up. The overall aim was to use cancer registry data to measure and improve the quality of cancer care. Now, 10 years later, the status of the extended cancer registration will be presented. In 2019, more than 500,000 new cancer cases were reported to the federal cancer registries. Age-standardised incidence has decreased slightly over the last decade. The five-year relative survival rate for all cancers was 67% for women and 63% for men. Therapy data show that an evidence-based assessment of quality of cancer care, including provider-based benchmarking, is feasible. Feedback of such results directly to healthcare providers should further improve cancer care. In conclusion, the introduction of population-based clinical cancer registration in Germany can be considered a success.

**Abstract:**

Introduction: In 2013, a new federal law obligated all German federal states to collect additional clinical data in population-based cancer registries as an active tool for monitoring and improving the quality of cancer care, increasing transparency and promoting health research. Now, 10 years later, the current status of the expanded cancer registration is presented, including current figures on cancer in Germany. Methods: Reporting of cancer is mandatory for physicians, and about 5 to 10 reports from different healthcare providers are expected for each case. A uniform national dataset of about 130 items is used, and reports are usually sent electronically to the registry. We used the most recent data available from cancer registries up to the year of diagnosis in 2019. We calculated incidence rates and 5-year relative survival (5YRS) for common cancers. Data on clinical outcomes and benchmarking based on quality indicators (QIs) from guidelines were provided by the Cancer Registry Schleswig-Holstein (CR SH). Results: All federal state cancer registries met most of the previously defined national eligibility criteria. Approximately 505,000 cancer cases were registered in 2019, with breast, prostate, colorectal and lung cancer being the most common cancers. The age-standardised cancer incidence has slightly decreased during the last decade. and spatial heterogeneity can be observed within Germany. 5YRS for all cancers was 67% and 63% for women and men, respectively. Therapy data for rectal cancer in 2019–2021 from the CR SH are shown as an example: 69% of the registered patients underwent surgery, mostly with curative intent (84%) and tumour-free resection (91%). Radiotherapy was given to 33% of the patients, and chemotherapy was given to 40%. Three selected QIs showed differences between involved healthcare providers. Discussion: The implementation of population-based clinical cancer registration can be considered a success. Comprehensive recording of diagnosis, treatment and disease progression and the use of registry data for quality assurance, benchmarking and feedback have been implemented.

## 1. Introduction

Cancer registration has a long history in Germany. The first cancer report was published in 1902, showing the prevalence of cancer at a regional level in Germany in cartographic form [1]. In 1927, the Hamburg Cancer Registry was established, followed by other regions in the following decades. In 1980, the German Childhood Cancer Registry (GCCR) was formed. However, it was not until 1994 that a federal law was passed, requiring all German federal states to establish population-based cancer registries (CRs). The law stated the following overarching goal: “Cancer registries shall monitor and evaluate cancer incidence and trends, provide data for epidemiological research, including causal research, and contribute to the evaluation of preventive and curative interventions” [2]. The law was implemented fairly quickly by most of the federal states [3,4], and a cancer registration system according to international standards [5] was established in Germany. In the Eastern German federal states, and in Bavaria, the structure of pre-existing hospital-based cancer registries was used for that purpose. Their registries already collected additional clinical data and information on therapies and relapse [6]. As this type of cancer registration has been successfully used for the quality assessment of oncological care, hospital benchmarking and accreditation, the 2008 German National Cancer Plan recommended the implementation of additional population-based registration of clinical data as an active tool to monitor and improve cancer care in Germany. In 2013, a new federal cancer registration law came into force, which now obliges all federal states to implement the additional registration of clinical data [4]. The superordinate goals of this law can be summarised under the terms of oncological quality assurance, transparency in cancer care and oncology health service research. At the same time, the law intended to facilitate national standardisation of the data to be collected; to ensure the exchange and pooling of the collected data in the complex German federal healthcare system; and finally, to provide guidelines for adequate funding.

It is now 10 years since the law came into force. Has the goal of comprehensive clinical registration been achieved? The aim of this paper is to describe the current status of extended cancer registration in Germany; to present recent analyses of cancer in Germany based, for the first time, on data with clinical content; and to evaluate developments over the past decade.

## 2. Materials and Methods

### 2.1. Aims and Tasks of the New Clinical Cancer Registration

In addition to the usual tasks of cancer registries, such as describing incidence, survival and trends, the additional clinical cancer registration has the task of monitoring and improving oncological care. The corresponding federal law [4] mentions the following points, among others, for this purpose:

- Evaluation of the collected clinical data and the feedback of the evaluation results to the individual healthcare providers;
- The promotion of interdisciplinary, directly patient-related cooperation in cancer treatment;
- Participation in the Joint Federal Committee’s inter-institutional and inter-sectoral quality assurance;
- Cooperation with centres in oncology;
- The provision of necessary data for the creation of transparency in care and for the purposes of healthcare research.

### 2.2. Structure of Cancer Registration

Germany currently has a population of 84 million inhabitants and consists of 16 federal states that are responsible for cancer registration (Table 1). The population size of the federal states varies between 680,000 and 18 million inhabitants. Each federal state has its own independent cancer registration structure, reflecting historical developments, with its own state law, budgetary sovereignty and registry topology. The exceptions are Berlin and Brandenburg, which form a geographical unit and operate a joint registry, and Saxony, which still has four separate regional sub-registries. The cancer registration laws of all federal states are comparable in content and aim to achieve uniform and interoperable cancer registration results throughout Germany. All federal and state laws on cancer registration have been aligned with the European General Data Protection Regulation (GDPR) [7]. In addition to the cancer registries of the federal states, two national institutions were established in the 1980s: The German Childhood Cancer Registry (GCCR), which records all cancers in persons younger than 18 years (the methods of the GCCR are described elsewhere [8]) and the German Centre for Cancer Registry Data at the Robert Koch Institute, which routinely collects data from the federal state registries to build a national cancer database [9]. Cancer registration is supported and harmonised at the national level by the following three associations: Association of Population-based Cancer Registries in Germany (GEKID, www.gekid.de (accessed on 5 June 2023)), Working Group of Tumour Centres (ADT, www.adt-netwerk.de (accessed on 5 June 2023) [8]) and the expert panel “Plattform 65c” for nationwide clinical cancer registration according to § 65c SGB V (www.plattform65c.de (accessed on 5 June 2023)).

### 2.3. Notification of Cancer and Data Processing

The notification of cancer cases is mandatory for all physicians and healthcare providers involved in the diagnosis or treatment of cancer. Five major events along the disease trajectory were defined by law as events requiring notification to the registry: diagnosis, pathology report, specific cancer therapy, disease progression or unremarkable follow-up or death, and optional tumour conference. At least three sets of information (diagnosis, pathology and therapy) should be available for a tumour. In practice, however, there are usually about 5–10 reports per tumour, as typically more than one healthcare provider is involved. With few exceptions, reports are submitted electronically to the registries, either by entering individual cases in an online portal or by uploading multiple cases from the tumour documentation software implemented at the reporting institutions. The basis for reporting is the uniform national and legally binding oncology dataset (Table 2), which is continuously reviewed and updated. It contains approximately 130 items for all types of cancer and currently four tumour-specific modules (colorectal, breast, prostate and melanoma) with supplementary items. In addition, regular transmission of death information and/or death certificates from civil registries and an exchange of notifications between cancer registries have been established. Multiple reports for a patient are linked using the unique health insurance number, name and address, and date of birth. If there is multiple information from different physicians on the same issue, the best information is determined according to national rules. A harmonised set of validity and plausibility checks is applied to the reported data. In the case of conflicting or insufficient information, a query back to the reporting physician is possible.

### 2.4. Funding of Cancer Registration

The new federal law radically changed the financing of population-based cancer registration. Whereas in the past funding was mainly provided by the federal states, 90% the operating costs of clinical cancer registration are now covered by the statutory health insurance funds and 10% are covered by the federal state. To receive this funding, cancer registries must meet a defined set of 43 eligibility criteria, including comprehensive indicators on data quality, such as completeness of registration (>90% of expected cases), completeness of the information to be reported, timeliness of registration, and reporting and feedback.

In 2023, the national reimbursement scheme for cancer registries has been set at approximately EUR 120 per incident and continuously documented case (according to IARC rules [5]) but may vary from state to state. On top of this, notifying physicians are compensated with payments of EUR 5–18 per notification, depending on the reporting event.

### 2.5. Data and Analyses

We used the most recent dataset with data from all federal cancer registries in Germany, provided by the German Centre for Cancer Registry Data at the Robert Koch Institute, Berlin (ZfKD). Data were available up to the year of diagnosis 2019. Absolute numbers, crude rates and age-standardised rates were calculated using the Segi world standard. Regional comparisons of age-standardised rates (European Standard, 1976) [10] and relative age-standardised 5-year survival rates (method: period analysis using the Ederer II approach [11]) were provided by the Cancer Atlas of the German Association of Population-based Cancer Registries (GEKID) [12], which also uses the most recent ZfKD dataset. Mortality data were extracted from the German Federal Statistical Office [13]. Nationally defined quality indicators (QIs) from evidence-based (S3) guidelines were used to assess guideline-compliant care [14,15]. To give an example on this data usage, the CR Schleswig-Holstein provided exemplary evaluations of quality indicators for three tumour entities (breast (ICD10 C50), colon and rectum (ICD10 C18-20) and prostate (ICD10 C61)) from the regional quality conferences of the year 2023. The degree of compliance with the QIs is shown as a bar chart for anonymised healthcare providers involved in cancer care. In addition, treatment data for rectal cancer (ICD10 C20) were extracted from the interactive treatment report of the CR Schleswig-Holstein [16]. All data used were retrieved in June 2023. Analyses were performed using SPSS 22 and R version 4.1.3.

## 3. Results

After the new federal law on the implementation of clinical cancer registries in the German states had come into force in 2013, all states have built up population-based registries that now meet the national eligibility criteria of full clinical cancer registration for several years. As a result, complete cancer registration coverage in terms of incidence has been achieved for the whole of Germany. In 2019, 505,612 incident cancer cases (C00-96, excluding C44) were registered by the 15 CRs of the federal states (Table 3). A total of 236,218 cases (46.7%) occurred in women (crude rate: 561.2/100,000), and 269,394 cases (53.3%*)* occurred in men (657.0/100,000). The most common cancer sites in women are breast (73,279), colorectal (26,655) and lung cancer (22,892), while prostate (70,192), lung (34,572) and colorectal (33,440) cancer were the most common cancers in men. The overall 5-year relative survival (5YRS) was 67% and 63% in women and men, respectively. The highest 5-year relative survival was observed for melanoma of the skin (95%), testicular cancer (92%), prostate cancer (90%), Hodgkin lymphoma (women 89%, men 86%), breast cancer (women 87%) and thyroid cancer (92%, 86%). The lowest 5YRS was observed for pancreatic cancer (14%, 12%), lung cancer (25%, 21%) and brain cancer (28%, 24%). Age-standardised rates for common cancers from 2015 to 2019 showed stable incidence rates in the last five years (Supplementary Table S1).

**Figure 1:**
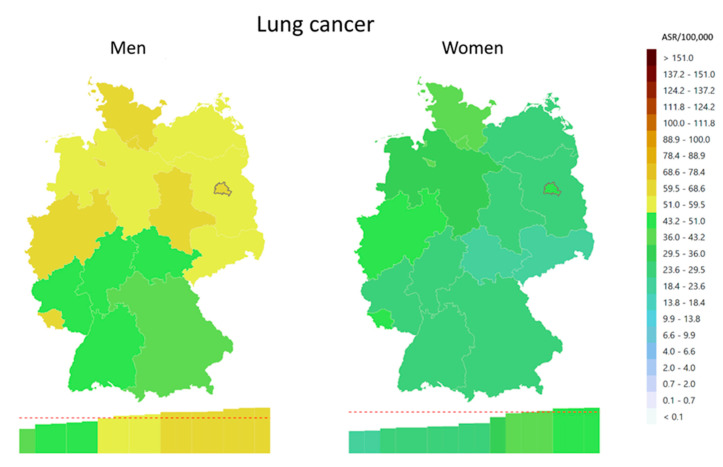
Geographical patterns of lung cancer incidence (ICD-10: C33-34) in 2019 in Germany on the level of federal states, age-standardised rates per 100,000 (ASR, European-Standard 1976). Data source: GEKID cancer atlas [12].

Regional differences in cancer incidence are present in Germany. For example, the age-standardised incidence of lung cancer in the region with the highest incidence is about twice that in the region with the lowest incidence (Figure 1). Other cancers with similarly large regional heterogeneity in incidence are cancers of the oral cavity and pharynx, liver, thyroid, and leukaemia and lymphoma. Common cancers like colorectal and prostate cancer show little heterogeneity. For breast cancer, lower rates are observed in the Eastern federal states (available online [12]).

**Figure 2:**
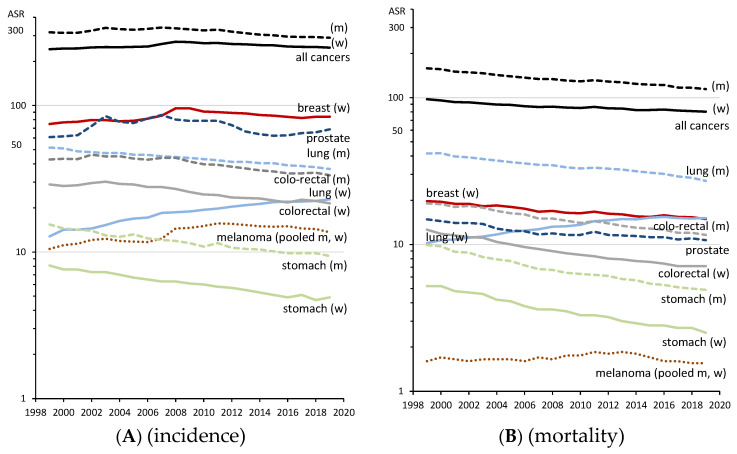
Incidence (**A**) and mortality (**B**) trends (1999–2019) of common cancers and all cancer sites (excluding C44), age-standardised rates per 100,000 (ASR, World). *Y*-axis logarithmic displayed. Data source: Centre for Cancer Registry Data at the Robert Koch-Institute [9].

Long-term trends of age-standardised cancer incidence show stable (men) or slightly increasing incidence for all cancer sites from 1999 until around the year 2008 (Figure 2A), followed by a slight decrease in the last decade (women: 272.6 to 248.3 per 100,000 (−9%); men: 336.7 to 290.4 per 100,000 (−14%), both from 2008 to 2019). This decline is mainly driven by colorectal cancer (−23%), stomach cancer (−22%) and lung cancer among men (−17%), whereas an increase is observed in lung cancer among women (+22%). Prostate cancer incidence is on the rise in the most recent years, after a maximum was reached in 2007, followed by a decline until 2015 (−23%). Cancer mortality (Figure 2B) has been declining for decades, except for lung cancer in women. Compared with 1999, the total cancer mortality in 2019 is 18% lower among women and 28% lower among men.

For the purpose of evaluating oncological care, all relevant treatment information is recorded (Table 2). Table 4 shows an extract of routinely published registry data on therapy, in this case for rectal cancer (C20), stratified by stage, based on a report from Schleswig-Holstein. For the interpretation, it should be noted that if no information is available for an item (missing or probably not reported data), it is assumed that the corresponding therapy was not carried out (conservative approach). Approximately 69% of patients underwent surgery, mostly with curative intent (84%). In sano resection (R0) was achieved in 91% of the cases, with the proportion in stages III–IV decreasing to 79% (vs. 86% in stages I–II). Radiotherapy was applied to 33% of the patients, but 4% of these patients discontinued therapy before the planned end of treatment due to patient rejection (*n* = 4), side effects (*n* = 12), progression (*n* = 1) or other reasons (*n* = 8). Chemotherapy was given to 40% of the patients, of which 44% received neoadjuvant chemotherapy therapy. The discontinuation rate was 22% (patient rejection (*n* = 9), side effects (*n* = 45), progression (*n* = 34) or other reasons (*n* = 73)). The most commonly used chemotherapeutic agents were antimetabolites (pyrimidine analogues as 5-FU, capecitabine, gemcitabine and cytarabine) (96%), platinum-based agents (48%) and topoisomerase inhibitors (16%). Immune/antibody therapy was administered to 12% of the patients.

**Figure 3:**
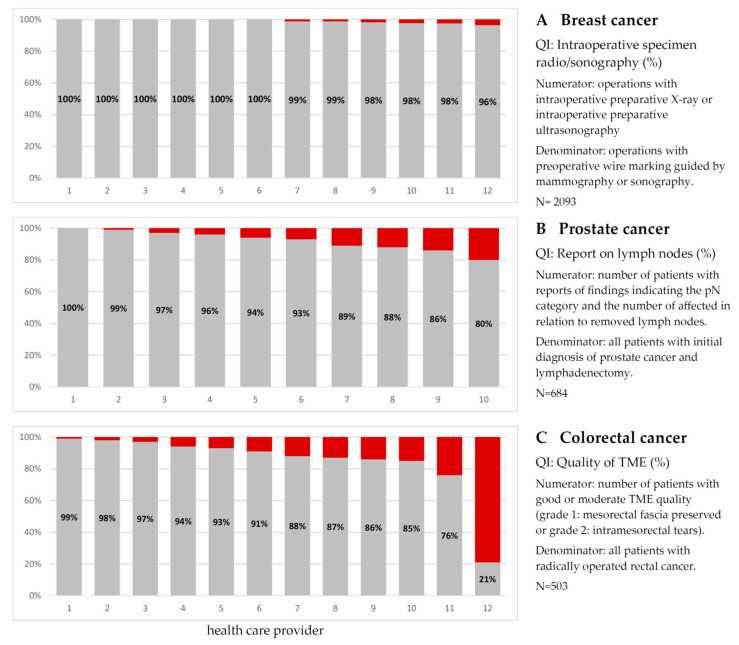
Selected quality indicators (QI), based on German S3 therapy guideline for patients diagnosed and treated in 2020/2021. Each bar reflects a single healthcare provider, involved in treatment with more than 40 cases per year. Data source: Cancer Registry of Schleswig-Holstein (www.cancer-sh.de (accessed on 5 June 2023)).

In addition to descriptive analyses of the therapeutic care provided to cancer patients, cancer registries are required to host regular regional quality conferences for healthcare providers. Quality indicators (QIs) defined in evidence-based (S3) guidelines are used to assess cancer care. Currently, about 50% (94/188) of these have been operationalised using CR data, including standardised definitions for the nominator and denominator of the QIs. Figure 3 shows an example of selected QIs for three tumour types, including the operationalisation of each QI. Each bar in the figure represents a healthcare provider, usually a hospital or larger outpatient facility. For patients with breast cancer (Figure 3A), interoperative radiography or ultrasound should be used whenever possible. All providers complied with this QI in 96% or more of the cases. For prostate cancer surgery, a qualified report on lymph node involvement should be provided (Figure 3B). Four out of ten providers did not reach 90%. For patients undergoing surgery for colorectal cancer (Figure 3C), the quality of the total mesorectal excision (TME) should be as high as possible. Six out of twelve providers scored above 90%.

## 4. Discussion

The introduction of clinical population-based cancer registration in Germany, initiated by a new law in 2013, can be considered a success. All major legal requirements have been implemented within the last 10 years. Although cancer registration for 84 million inhabitants is carried out by 15 independent cancer registries, decentralised registration is feasible and a high degree of standardisation in cancer registration has been achieved. Successful measures include not only uniform registration with a mandatory standardised dataset but also data exchange between registries, common standards for evaluation, and national availability of data for health monitoring and research. In this respect, cancer registration has now reached a level comparable with that of large international programmes such as SEER [17]. Such a major change in cancer registration from a dataset with only few recorded items, partly submitted as paper-based records, to full electronic recording with an extensive dataset, could have led to breaks in the epidemiological key figures. Luckily, this conversion to clinical cancer registration has not led to any breaks or temporal gaps in completeness or data quality. Time trends in incidence and other epidemiological indicators show stable values. On the contrary, the transition has improved the national completeness of cancer registration. In previous years, incidence had to be estimated for the whole of Germany due to regional registration gaps [18], but now, cancer incidence can be simply counted and reported on the basis of reported cancer cases. A comparison of cancer incidence in Germany with that of its northern neighbours, the Scandinavian countries, shows similar values and trends [19]. In both regions, the total cancer incidence (excluding non-melanoma skin cancer) decreased slightly in recent years. In 2019, the age-standardised incidence (world) in the Scandinavian countries was 288 and 311 per 100,000 for women and men, respectively, which is comparable with the figures for Germany (249/290 per 100,000 women/men). The same applies to the comparison with the western neighbour, the Netherlands, where the incidence in 2019 was 293/303 per 100,000 women/men [20]. Also, the European Cancer Observatory estimates for Germany (ECIS: 267/307 per 100,000 women/men) [21] are in concordance with our reported findings. Overall, the population-based approach of German cancer registration seems to provide reliable results on national level. Survival data are also in line with European results, as provided by ECIS. At this point it should be mentioned that the complete data of the cancer registries, partly going back to 1967, are accessible for research and have already been used intensively. The registry data are also regularly used for health reporting. There are interactive databases for incidence, mortality and survival at the federal and state levels up to small-area cancer reporting at the level of municipalities [22].

The most important advancement in cancer registration is the use of CR data for quality assurance and quality management. Treatment, progression and survival outcomes are now considered not only at the regional population level but also at the micro level of individual healthcare providers (HCPs). This includes not only feedback to individual HCPs on the quality of their own processes and outcomes but also benchmarking of the quality of care on a provider-by-provider basis. At present, such comparisons are mainly used to discuss the quality of cancer care with HCPs behind closed doors, without the public being present. However, the first countries have begun to publish quality parameters and name the institutions [23]. This is a big step towards transparency in oncology, and it is likely that this level of transparency will be introduced in other regions. In the long term, this may enable patients to choose their HCP on the basis of quality. It should be emphasised that the assessment of quality of care by cancer registries is not limited to selected hospitals but covers the entire oncological care landscape. This is particularly important for Germany with its strictly segmented healthcare system (strict separation of treatment in hospitals and outpatient/office-based care). The quality of care at cancer centres, which undergo voluntary annual certification by the German Cancer Society, is well known (see annual reports for different cancer types [24]). However, it is estimated that only 50% of all cancer patients are treated in these facilities (own calculation based on [24]), so that the quality of cancer care for all cancer cases is still unknown. Population-based cancer registries, which record the care of cancer patients in all settings (inpatient and outpatient), allow a holistic assessment of cancer care. A recent publication by the European Network of Cancer Registries shows that about 50% of European cancer registries also collect clinical data, but the available data on therapy appear to be crude, such as surgery or chemotherapy yes/no [25], and few registries collect the clinical data needed to assess the quality of care [26]. There are several publications comparing accredited hospitals on the basis of quality indicators, for example for breast cancer [27] or renal cancer [28], but these reports are based on specific hospital-based data collection and not on routine cancer registration. However, recent publications indicate that the use of CR data for quality assessment at the HCP level is just beginning. In the Netherlands, hospital comparisons based on quality indicators have already been published for gynaecological cancers [29,30,31], lung cancer [32] or rectal cancer [33]. Such analyses, like ours, show that cancer care can be made measurable at the HCP level and that fair benchmarking of specific clinical aspects is possible. However, only the future will tell whether monitoring quality of care will ultimately lead to improved patient-relevant clinical outcomes. Do HCPs with well-documented quality produce better clinical outcomes than HCPs with poorly documented quality? Do recurrence rates decrease with increasing quality, do survival rates or quality of life improve? Cancer registry data will help answer such questions in the future.

It should be noted that all these efforts require a high financial input. In Germany, about EUR 160 is spent per registered case (EUR 120 for the CR + EUR 40 for five notifying physicians), or even more if relapse and additional therapies are reported, which is significantly higher than the European average of about EUR 50 per case, as reported by Zanetti et al. in 2014 [34]. A rough estimate—500,000 incident cancers per year, five notifications each—results in a sum of about 80 million EUR per year for Germany. 90% of these costs are covered directly by the health insurance funds. This has been facilitated by the fact that the new law makes cancer registration an integral part of public healthcare [35]. For the same reason, the use of registry data for quality assurance purposes became possible, independent of the consent of the institution. As a kind of return on investment, the healthcare system (and health insurance funds) obtains a comprehensive, evidence-based view of oncological care in order to initiate specific HCP-based measures for improvement, if necessary.

Limitations and outlook: Although much has been achieved in the last 10 years and cancer registration has undoubtedly reached a new level, major efforts are still needed to achieve all goals. For example, although it can be assumed that all incident cancers are currently fully recorded in cancer registries, data on treatment are not yet fully available. This is partly due to the sectoral structure of the German healthcare system, partly due to the fact that patients in Germany have complete freedom of choice of doctor or hospital, and partly due to the fact that the technical requirements for complex CR reporting and the corresponding software are still being developed. The latter limitation will be overcome in the next few years through increasing national efforts in digitisation and interoperability.

The national pooled dataset available for research purposes at the ZfKD will be expanded to include core clinical variables on treatment and disease progression based on the new federal law for data consolidation form the year 2021 [36]. The law paves the way to easier access to CR data for research, data linkage with other health data, identification of similar disease cases (digital twin) or long-term effects of cancer. In addition, the inclusion of patient-reported outcomes and patient-reported experience measures in cancer registries is being discussed as direct patient involvement in the registration process. In the near future, first results are expected to show whether the additional clinical cancer registration not only improves the process quality of oncological care but also leads to patient-relevant improvements regarding clinical outcome quality.

## 5. Conclusions

The implementation of clinical, population-based cancer registration can be considered a success. Basic features such as comprehensive recording of diagnosis, treatment and disease progression or the use of registry data for quality assurance, benchmarking and feedback have been implemented over the last 10 years. However, considerable efforts are still needed to achieve the goal of comprehensive and transparent assessment of oncological care in Germany.

## Figures and Tables

**Table 1:** Federal state cancer registries (CR) in Germany and national cancer registry structures.

	Population (Mio)	Founded	Clinical Data Since
CR Baden-Wurttemberg	11.1	1994	2009
CR Bavaria	13.1	1998	2017
CR Berlin and Brandenburg	6.0 (3.7/2.3)	1953	1953
CR Bremen	0.7	1998	2015
Hamburg CR	1.9	1927	2014
CR Hesse	6.3	2001	2015
CR Lower Saxony	8.0	2000	2018
CR Mecklenburg-Western Pomerania	1.6	1953	1953
CR North Rhine-Westphalia	17.9	** 1986	2016
CR Rhineland-Palatinate	4.1	1997	2016
Saarland CR	1.0	1968	2016
CR Saxony	4.1	1953	1953
CR Saxony-Anhalt	2.2	2018	1993
CR Schleswig-Holstein	2.9	1997	2017
CR Thuringia	2.1	1953	1953
German Childhood Cancer Registry	13.5	1980	-
German centre of cancer registry data at the Robert Koch-Institute *	(84.0)	1983	2023

* data provided by federal state CRs; ** administrative district of Münster within North Rhine-Westphalia; since 2005 full coverage of North Rhine-Westphalia.

**Table 2:** Simplified description of the mandatory German basic oncology dataset and its modules. Detail version available from https://basisdatensatz.de/basisdatensatz (accessed on 5 June 2023).

Item Class	Collected Information
*Basic dataset*	~130 item
Personal data patient	Health insurance numbers, names, address, date of birth and gender
Personal data notifying institution	Names, address, billing data
Cancer diagnosis	ICD-10, ICD-O topography, date, certainty, side
Histology	Date, ICD-O morphology, grading, lymph nodes examined and involved, if pathology report: name and address of physician sending in the sample
Tumour classification	TNM and other
Genetic variants	Type
Residual state	After surgery and total assessment
Performance status	ECOG (at diagnosis)
Surgery	Date, intention, OPS codes, complication
Radiotherapy	Date, intention, position to surgery, target area, begin, end, dose, boost, complication (CTCAE)
Systemic therapy	Date, intention, position to surgery, kind of therapy (including active surveillance), protocol, drug, begin, end
Tumour conference	Date, type
Follow-up	Date, total assessment of tumour, lymph nodes, metastases
Death	Date, cause
*Organ specific modules*	Specific items for quality assurance
Breast cancer module	10 items
Prostate cancer module	10 items
Colorectal cancer module	12 items
Melanoma of the skin module	4 items

**Table 3:** Most common cancer sites (ICD-10) in Germany in 2019: case numbers (N), crude rates per 100,000 (CR), age-standardised rates per 100,000 (ASR, World standard) and relative 5-year survival (5Y-RS) (period 2017–2019), percentage of all cancers (%) and ranking (place). Data source: common dataset of German cancer registries provided by the Centre for Cancer Registry Data at the Robert Koch-Institute, 2023.

	Women					Men				
Cancer Site	N	CR	ASR	% (Place)	5Y-RS%	N	CR	ASR	Place-S	5Y-RS%
Oral cavity, pharynx (C00–C14)	4357	10.4	4.6	1.8 (15)	61.3	9521	23.2	11.5	3.5 (9)	50.5
Oesophagus (C15)	1627	3.9	1.5	0.7 (19)	26.3	5694	13.9	6.3	2.1 (12)	26.4
Stomach (C16)	5650	13.4	4.8	2.4 (10)	39.1	9091	22.2	9.4	3.4 (10)	35.0
Colo-rectal (C18–C20)	26,655	63.3	22.1	11.3 (2)	68.2	33,440	81.6	34.3	12.4 (3)	64.8
Pancreas (C25)	9546	22.7	7.3	4.0 (7)	13.6	9614	23.4	9.6	3.6 (7)	12.4
Lung (C34)	22,892	54.4	22.5	9.7 (3)	25.0	34,572	84.3	36.0	12.8 (2)	20.6
Melanoma (C43)	11,038	26.2	14.1	4.7 (5)	95.3	12,527	30.6	14.4	4.7 (5)	94.0
Breast (C50)	73,279	174.1	86.2	31.0 (1)	86.5	721	1.8	0.8	0.3 (18)	78.8
Cervix uteri (C53)	4517	10.7	7.2	1.9 (14)	61.6	-	-	-	-	-
Corpus uteri (C54–C55)	11,221	26.7	11.6	4.8 (4)	78.4	-	-	-	-	-
Ovary (C56)	7128	16.9	7.6	3.0 (9)	41.3	-	-	-	-	-
Prostate (C61)	-	-	-	-	-	70,192	171.2	70.0	26.1 (1)	90.3
Testis (C62)	-	-	-	-	-	4113	10.0	9.3	1.5 (14)	92.4
Kidney (C64)	4912	11.7	4.6	2.1 (12)	79.2	9505	23.2	10.9	3.5 (8)	78.2
Bladder (C67)	4697	11.2	3.5	2.0 (13)	70.4	13,116	32.0	12.2	4.9 (4)	78.5
Brain (C70–C72)	3002	7.1	4.0	1.3 (18)	27.9	3909	9.5	5.5	1.5 (13)	24,3
Thyroid gland (C73)	4113	9.8	7.3	1.7 (16)	92.0	1816	4.4	3.0	0.7 (16)	86.3
Hodgkin lymphoma (C81)	1063	2.5	2.3	0.5 (20)	89.1	1386	3.4	2.7	0.5 (17)	86.0
Non-Hodgkin lym. (C82–C88)	8094	19.2	7.7	3.4 (8)	73.3	10,042	24.5	11.3	3.7 (6)	71.1
Multiple myeloma (C90)	3073	7.3	2.5	1.3 (17)	59.3	3835	9.4	3.9	1.4 (15)	57.8
Leukaemia (C91–C95)	5263	12.5	5.2	2.2 (11)	59.0	7467	18.2	8.5	2.8 (11)	60.7
All sites excluding C44	236,218	561.2	249.0	100.0	67.0	269,394	657.0	290.3	100.0	63.4

**Table 4:** Treatment of rectal cancer (ICD-10 C20) patients, treated in 2019–2021 in Schleswig-Holstein, stratified by tumour stage. Data source: interactive cancer report CR Schleswig-Holstein [16].

	Patients Total N = 1734	UICC Stages I–II N = 554	UICC Stages III–IV N = 792	UICC Stage Unknown N = 388
*Surgery*	1200 (69%)	432 (78%)	591 (75%)	177 (46%)
Curative intention	1003 (84%)	372 (86%)	465 (79%)	166 (94%)
Resection R0 *	1047 (91%)	394 (95%)	496 (87%)	157 (92%)
Operation within 14 days after diagnosis	285 (24%)	128 (30%)	145 (25%)	12 (7%)
*Radiation*	850 (33%)	92 (17%)	286 (36%)	202 (52%)
Curative intention	552 (95%)	90 (98%)	268 (94%)	194 (96%)
Premature termination	25 (4%)	5 (5%)	11 (4%)	9 (4%)
Neoadjuvant	452 (78%)	67 (73%)	215 (75%)	170 (84%)
*Chemotherapy*	698 (40%)	87 (16%)	428 (54%)	183 (47%)
Curative intention	458 (69%)	68 (72%)	280 (69%)	110 (65%)
Premature termination	152 (22%)	10 (11%)	1127 (26%)	30 (16%)
Neoadjuvant	310 (44%)	41 (47%)	175 (41%)	94 (51%)
Immune/Antibody-therapy	99 (14%)	2 (2%)	79 (18%)	18 (10%)
cytostatic agents used:				
Antimetabolic	672 (96%)	85 (98%)	410 (96%)	177 (97%)
Platin-based	333 (48%)	29 (33%)	217 (51%)	110 (60%)
Topoisomerase inhibitors	115 (16%)	2 (2%)	90 (21%)	23 (13%)

* R0, no residual tumour/in sano, R-classification.

## Data Availability

All used data were extracted from public or freely accessible databases. Data sources are included in the references.

## Supplementary Materials

The following supporting information can be downloaded at: https://www.mdpi.com/article/10.3390/cancers15153934/s1, Table S1: Most common cancer sites (ICD-10) in Germany 2015 to 2019: age-standardized rates per 100,000 (ASR World) Data source: common data set of German cancer registries provided by the Centre for Cancer Registry Data at the Robert Koch-Institute, 2023.
