# Supplementary material for: Population-Based Clinical Cancer Registration in Germany
Source: Cancers (Basel). 2023 Aug 2;15(15):3934. doi: 10.3390/cancers15153934 (PMC10416989; doi:10.3390/cancers15153934)
Supplement: Supplementary file 1 [file cancers-15-03934-s001.zip › cancers-2510992-supplementary.pdf]

Supplementary Material

Table S1: Most common cancer sites (ICD-10) in Germany 2015 to 2019: age-standardized rates per 100,000 (ASR World)

Data source: common data set of German cancer registries provided by the Centre for Cancer Registry Data at the Robert Koch-Institute, 2023

	**Women**					**Men**				
Cancer site	2015	2016	2017	2018	2019	2015	2016	2017	2018	2019
Oral cavity, pharynx (C00-C14)	4.6	4.4	4.6	4.7	4.6	12.6	12.5	12.3	11.9	11.5
Oesophagus (C15)	1.5	1.5	1.5	1.5	1.5	6.5	6.6	6.3	6.1	6.3
Stomach (C16)	5.1	4.9	5.2	4.8	4.8	10.1	9.8	9.9	9.7	9.4
Colo-rectal (C18-C20)	22.4	22.0	23.0	23.1	22.1	35.6	34.6	34.9	35.3	34.3
Pancreas (C25)	7.2	7.3	7.4	7.2	7.3	9.7	9.9	9.6	9.8	9.6
Lung (C34)	21.6	21.7	22.1	22.1	22.5	39.5	38.6	38.1	37.2	36.0
Melanoma (C43)	15.5	15.4	14.9	14.6	14.1	14.8	14.7	14.4	14.6	14.4
Breast (C50)	86.3	85.3	84.8	85.6	86.2	0.8	0.8	0.7	0.7	0.8
Cervix uteri (C53)	7.3	7.2	7.3	7.2	7.2	-	-	-	-	-
Corpus uteri (C54-C55)	11.4	11.4	11.5	11.6	11.6	-	-	-	-	-
Ovary (C56)	8.5	8.0	8.0	8.0	7.6	-	-	-	-	-
Prostate (C61)	-	-	-	-	-	61.3	63.0	66.0	67.3	70.0
Testis (C62)	-	-	-	-	-	9.3	9.0	8.8	9.4	9.3
Kidney (C64)	5.3	5.3	4.9	5.0	4.6	11.6	11.1	11.0	10.8	10.9
Bladder (C67)	3.4	3.5	3.6	3.5	3.5	12.4	12.3	12.2	12.4	12.2
Brain (C70—C72)	4.6	4.7	4.3	4.2	4.0	6.1	6.1	5.7	5.8	5.5
Thyroid gland (C73)	8.2	8.2	8.1	7.6	7.3	3.2	3.3	3.2	3.0	3.0
Hodgkin lymphoma (C81)	2.3	2.0	2.1	2.2	2.3	2.7	2.8	2.8	2.8	2.7
Non-Hodgkin lym. (C82-C88)	7.9	8.0	8.0	7.9	7.7	11.6	11.6	11.6	11.2	11.3
Multiple myeloma (C90)	2.7	2.6	2.7	2.5	2.5	4.1	4.2	4.1	3.9	3.9
Leukaemia (C91-C95)	6.2	6.1	5.8	5.4	5.2	9.9	9.5	9.3	8.8	8.5
All sites excluding C44	255.1	252.0	252.3	251.4	249.0	295.2	292.8	292.6	292.4	290.3
